# Aerosolized avian influenza virus by laboratory manipulations

**DOI:** 10.1186/1743-422X-9-146

**Published:** 2012-08-06

**Authors:** Zhiping Li, Jinsong Li, Yandong Zhang, Lin Li, Limin Ma, Dan Li, Feng Gao, Zhiping Xia

**Affiliations:** 1 College of Animal Science and Veterinary Medicine, Jilin University, Changchun, 130062, China; 2 Key Laboratory of Jilin Province for Zoonosis Prevention and Control, Veterinary Research Institute, Academy of Military Medical Sciences, Changchun, 130122, China; 3 Insititute of Microbiology and Epidemiology, Academy of Military Medical Sciences, Beijing, 100071, China; 4 Department of Rheumatology, First Hospital, Jilin University, Changchun, 130021, China

**Keywords:** H5N1, Aerosol, Laboratory-associated infections, Occupational and environmental safety

## Abstract

**Background:**

Avian H5N1 influenza viruses present a challenge in the laboratory environment, as they are difficult to collect from the air due to their small size and relatively low concentration. In an effort to generate effective methods of H5N1 air removal and ensure the safety of laboratory personnel, this study was designed to investigate the characteristics of aerosolized H5N1 produced by laboratory manipulations during research studies.

**Results:**

Normal laboratory procedures used to process the influenza virus were carried out independently and the amount of virus polluting the on-site atmosphere was measured. In particular, zootomy, grinding, centrifugation, pipetting, magnetic stirring, egg inoculation, and experimental zoogenetic infection were performed. In addition, common accidents associated with each process were simulated, including breaking glass containers, syringe injection of influenza virus solution, and rupturing of centrifuge tubes. A micro-cluster sampling ambient air pollution collection device was used to collect air samples. The collected viruses were tested for activity by measuring their ability to induce hemagglutination with chicken red blood cells and to propagate in chicken embryos after direct inoculation, the latter being detected by reverse-transcription PCR and HA test. The results showed that the air samples from the normal centrifugal group and the negative-control group were negative, while all other groups were positive for H5N1.

**Conclusions:**

Our findings suggest that there are numerous sources of aerosols in laboratory operations involving H5N1. Thus, laboratory personnel should be aware of the exposure risk that accompanies routine procedures involved in H5N1 processing and take proactive measures to prevent accidental infection and decrease the risk of virus aerosol leakage beyond the laboratory.

## Background

The pandemic H1N1 outbreak of 2009 and global threat of H5N1 in recent years have been accompanied by a large amount of laboratory-based experimental research activity using purified viruses and infected tissues and animals. The majority of these studies have focused on either natural infections from the community or induced infections in the laboratory, with very few studies considering the infection risk or outcome of laboratory personnel handling the samples. The potential of an accidental laboratory-acquired infection is well-recognized among laboratory staff and researchers. In 1941, Meyer and Eddie published the first report of laboratory infections due to the Gram-negative bacteria Brucella [1]. In 1949, Sulkin and Pike published a report that summarized 222 laboratory-acquired infections due to viruses [2]. Since then, significant efforts have been made by the oversight committees of research institutes and governmental bodies to establish occupational and environmental safety guidelines to protect workers and the local community alike from laboratory-acquired infections; however, these infections have yet to be eradicated and many have been reported over the past eight decades [3-9].

The total number and relative frequency of bacterial laboratory-acquired infections has, in fact, declined dramatically over time [10-12]. In contrast, the relative frequency of viral laboratory-acquired infections has increased by 60% [13]. Research into the underlying factors responsible for these infections have indicated that the main route of infection, for both bacteria and viruses [9,14-20], is through mucous membranes that are contaminated by inhaling pathogens, not dissimilar from the natural route of infection [21]. Traditionally, the risk of laboratory infection has been minimized by simply practicing good laboratory practice (GLP), which is otherwise necessary for reliability of the laboratory work itself. A detailed examination of the publically available information on all reported laboratory-acquired infections, however, indicated that ~80% were not the result of overt "accidents"; it is, thus, likely that inhalation of aerosolized infectious particles that are liberated by normal laboratory techniques account for a large portion of laboratory-acquired infections.

Research into this theory has indicated that influenza virus transmission and infection can be achieved through aerosols [22]. Many of the routine procedures used to process influenza virus for laboratory research, such as centrifugation or mixing, have a high potential of producing aerosols [21], and the particle load of each has been estimated to be up to 1–5 μm. In addition, it is expected that larger particles will tend to fall out of the air and contaminate surfaces, by which individuals may be infected by contact or may transmit the particles to a secondary aerosol [4]. Fundamentally, aerosols are suspensions in the air of solid or liquid particles small enough that they will remain transmissible and airborne for a prolonged period of time [23]. Particles of 5 μm or less increase the risk of establishing an infection upon airborne transmission, as they are remarkably capable of penetrating the physical cellular barrier of the respiratory tract and traveling all the way to the alveolar region. As with naturally-acquired infections, most individuals are not diagnosed before onset of symptoms, impeding the time to initiation of treatment [24].

The A type influenza viruses are commonly spread by the airborne route in normal circumstances. Accordingly, more research on the potential and character of aerosol spread of influenza virus has been carried out [25-27], and many studies have used experimental animal models of aerosol infection to mimic the natural process [19,28,29]. However, less information is available on the features of laboratory-produced aerosolized influenza virus.

The Australian researcher, Adrian Gibbs, suggested that the A/H1N1 flu virus currently circulating around the world was created in a laboratory. Although the World Health Organization (WHO) eventually dismissed this theory, suspicion and panic were aroused in the general public about laboratory safety. There are many situations that may facilitate the spread of a pathogen from the laboratory, ranging from aerosols produced by routine procedures or misuse of laboratory equipment to uncontrollable natural disasters that impact the structural integrity of the laboratory, such as earthquake or fire. In order to regulate the potential of pathogen transmission from the laboratory, we must first gain a detailed understanding of the experimental operations that produce aerosols. To this end, this study was designed to monitor the presence of aerosolized H5N1 virus produced by normal procedures used to process the virus for experimental research and by the most frequently associated “accidents” for each, such as container breakage and accidental subcutaneous injection. This information will help to guide future experimental practice standards to ensure the safety of laboratories and laboratory personnel, thereby increasing community confidence in laboratory bio-safety.

## Results

### Experimental application of the aerosol collection device

**Figure 1:**
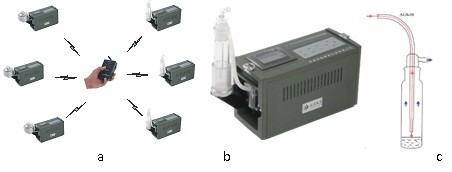
**Micro-cluster sampling ambient air pollution collection device.** The device consists of a controller and six pumps with AGI-30 or Andersen **(a)**. The device relies on the liquid impactor approach to capture airborne viruses **(b)**. Red, thin lines and arrows represent the airflow into the sampler. Blue, thick arrows represent airflow out of the sampler **(c)**. The cartoon is a simplified representation.

The prototype sampling device, shown in Figure 1, consisted of a controller and six pumps with AGI-30 or Andersen impingers of a wireless networking technology. This instrument can collect several samples of aerosols simultaneously, or can be set to have one of the six pumps operate independently. We used liquid impingers, which rely on inertial collection mechanisms to collect aerosolized particles [30] and were situated as described in the Materials and Methods. The sampler outlet was situated above the nozzle outlet; thus, there was a sharp turn in flow streamlines at the nozzle outlet, just above the liquid surface. Particles with high inertia cannot follow sharp turns in streamlines and will impact and penetrate the liquid surface after exiting the nozzle.

### Result of normal operation of laboratory procedures

At the start of each group experiment, the sampler was operated for 30 minutes to obtain baseline readings of the laboratory atmosphere. Collection of aerosols from the seven groups of experimental procedures and controls followed the steps detailed in the Materials and Methods section. The processing time was 30 min for each group, except for group I (time was 1 h), and the air sampling distance was 38 cm from the working materials. Statistics of the group temperature and relative humidity for each of the data collection processes, including operations and control of the collection, were measured automatically by the aerosol collector.

**Figure 2:**
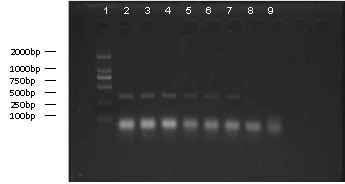
**Result of Rt-PCR for aerosol samples from each group.** Lanes: 1, marker; 2, zootomy group; 3, grinding group; 4, pipetting group; 5, magnetic stirring group; 6, egg inoculation; 7, experimental zoogenetic infection group; 8, centrifugal group; 9, control group.

None of the aerosol samples collected from any group at the time directly prior to processing of the experiment had detectable levels of H5N1, as evidenced by negative reverse transcription (Rt)-PCR and HA text results. All liquid aerosols collected from the experimental procedure of group III were also negative for H5N1 (Table 1, Figure 2). The aerosols collected during the experimental procedures of all other six groups were positive for H5N1. The aerosols from each group taken after disinfection were negative (Table 2).

**Table 1:** HA text detection of H5N1 in aerosols generated

	**Zootomy**	**Grinding**	**Pipetting**	**Magnetic stirring**	**Egg inoculation**	**Zoogenetic infection**	**Broken glass**	**Syringe-ejected influenza virus suspension**	**Centrifuge tube rupture**
**HA Text**	2^5^	2^5^	2^7^	2^7^	2^8^	2^9^	2^9^	2^10^	2^10^

**Table 2:** Rt-PCR detection of H5N1 in aerosols generated by various experimental procedures

	**Group I: zootomy**	**Group II: grinding**	**Group III: centrifugation**	**Group IV: pipetting**	**Group V: magnetic stirring**	**Group VI: egg inoculation**	**Group VII: zoogenetic infection**
**Collection time, h**	1	0.5	0.5	0.5	0.5	0.5	0.5
**Temperature,°C**	29.5 ± 3.6	30.6 ± 1.7	28.1 ± 1.0	25.2 ± 1.9	28.0 ± 2.1	30.8 ± 0.9	30.9 ± 1.9
**Relative humidity,%**	28.0 ± 5.1	22.8 ± 2.2	25.6 ±1.3	30.5 ± 3.3	26.1 ± 3.2	22.3 ± 1.3	21.9 ± 2.4
**Positive experimental samples, n/total**	6/6	6/6	0/3	3/3	3/3	3/3	3/3
**Positive of control samples, n/total**	0/6	0/6	0/3	0/3	0/3	0/3	0/3

### Result of laboratory procedures operation with simulated accidents

**Figure 3:**
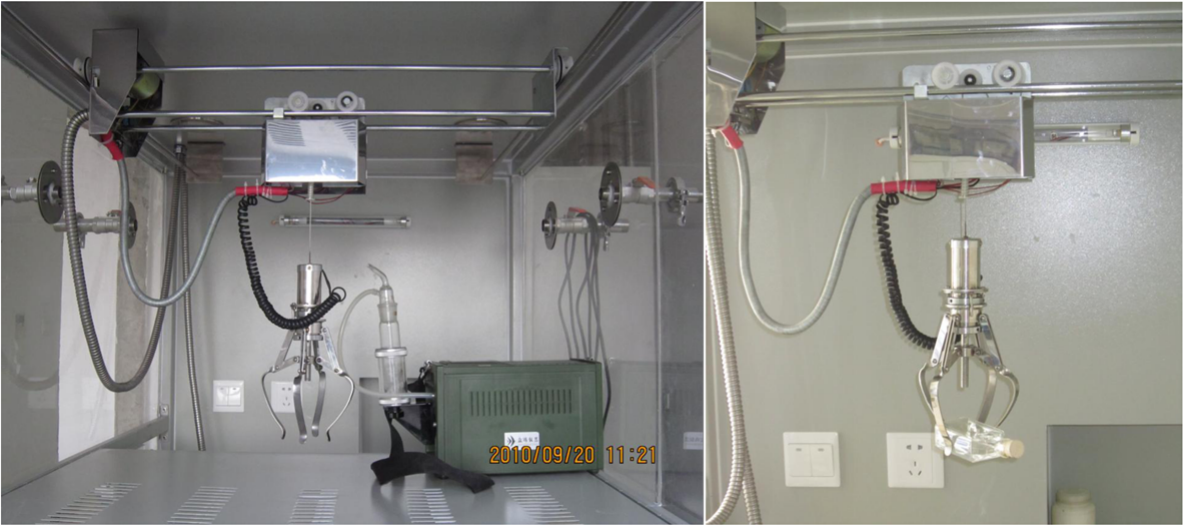
**Modified negative pressure isolation unit with a mechanical arm.** Modified negative pressure isolation unit with a mechanical arm to carry out controlled ‘accident’ procedures generating virus-carrying aerosols,

Our experiment was carried out in a negative pressure isolation unit with mechanical arm (Figure 3).

**Figure 4:**
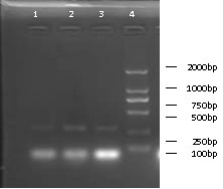
**Rt-PCR analysis of the procedures operation with simulated accidents. ** Lanes: 1, broken glass container group; 2, syringe-ejected influenza virus suspension group; 3, tube rupture group.

The three groups of procedures with simulated accidents (broken glass containers with influenza virus suspension, syringe-ejected influenza virus suspension, and centrifuge tube rupture) all produced aerosols. The controls for each were all negative. All of the aerosols that were produced contained H5N1 that was detectable by Rt-PCR and HA text (Table 1, Figure 4).

## Discussion

The field of laboratory-acquired viral infections has progressed since Sulkin and Pike’s seminal report in 1949 [2], but the problem has yet to be completely eliminated. The paucity of experimental data is an important reason for this. Our study, presented herein, provides the first evidence of laboratory procedures that generate viral contamination that can potentially infect laboratory personnel.

In fact, all of the studies performed to date by others to determine whether laboratory studies of pathogens, including viruses, bacteria and fungi, could generate infective aerosols have been based on statistical analysis of actual infections. The influenza viruses present a particular challenge to monitoring in the lab by traditional methods, since their small size and low concentration make them difficult to collect and detect from the air. Most of the bioaerosol samplers currently on the market are not suitable for collection of viruses [31]. Furthermore, effective detectors need to be able to conveniently collect air samples at various time points over the course of an entire experiment since virus-containing aerosols may be generated by any number of steps in the process and then fall from the air prior to an end-of-procedure sampling time point. The currently available samplers typically run for short periods of time (minutes), making it difficult to capture large volumes or integrate sample collection over time. We designed a collector terminal with six air pollution sampling probes that are able to continuously sample the air for ≥10 h; in addition, this device is portable and can be easily moved around the laboratory to locales where different steps of the experiment are performed. The feature of remote control allows for simultaneous or sequential collection in different locales from two or more of the probes. In the study described herein, to test the capability of such a detector, the aerosols collected during the experiment reflected the virus produced in most operations; this study was not designed to clearly delineate a complete picture of all potential applications of this device.

Measuring infectious virus from air samples is logistically difficult. Few researchers have reported airborne influenza virus from the laboratory, and even fewer have detected the infectious capacity of influenza viruses sampled from air. Traditional methods of determining the presence of virus in an aerosol include directly applying the concentrated aerosol to infect a host cell, or as template in Rt-PCR to detect virus-specific genes. One of the most commonly used methods is Rt-PCR because of its remarkable sensitivity and specificity; however, Rt-PCR is unable to determine whether the detected virus is infectious. In our study, we combined experimental approaches that would determine both the concentration of virus in aerosols and the activity of those viruses. By exploiting the functional character of the hemagglutinin of influenza virus, we were able to estimate the concentration of influenza virus in aerosols according to the amount of adsorption that occurred with red blood cells (RBCs). By also using the aerosols to directly inoculate chick embryos, we were able to determine the ability of the aerosolized virus to proliferate *in vivo*, indicating the infectivity of the contaminating pathogens. Future studies will aim to determine the feasibility of this approach for quantifying virus in the aerosol.

This study, to our knowledge, represents the first successful attempt to directly detect influenza virus-containing aerosols generated by routine laboratory procedures. Our results provide evidence that many of the laboratory techniques used to process influenza virus for experimental analysis produce aerosols and, thereby, represent significant risks of infection to laboratory personnel and potential spread beyond the laboratory. Working in the laboratory is inevitably dangerous, but nearly all risks can be sufficiently minimized by GLP and careful monitoring of risk factors, such as presence of pathogen-containing aerosols. According to the results of this study, we plan to extend our investigations to emergency operating procedures that follow laboratory accidents with virus samples and to generate more effective strategies to prevent laboratory-generated aerosols and laboratory-acquired infections or spread.

## Conclusions

In summary, our findings demonstrated that many of the laboratory techniques used to process influenza virus produce aerosols, either through normal operations or common accidents associated with each process. Our results have great value and implications towards bio-safety and future strategies to evaluate risks of experimental virology to laboratory workers and the general public.

## Materials and methods

### Virus

Purified avian influenza virus (A/chicken/Jilin/9/2004(H5N1), GenBank: AY653193 ~ AY653200) was obtained from our laboratory stock, and diluted 10^-1^ in phosphate buffered saline (PBS, 0.1 mol/L) supplemented with free calcium and magnesium. Aliquots of this stock solution were stored at −80°C until use. Prior to experimentation, virus was propagated by infection in chicken eggs, and yield was determined to be 6.7 log10 50% egg infection doses per milliliter (EID50/mL). All the experiments on animals were approved by the Ethics Committee of Animal Science and Veterinary Medicine of Jilin University, First Hospital of Jilin University and Academy of Military Medical Sciences, in accordance with guidelines of the Nation Health and Medical Research Council of China.

### Aerosol collection

Aerosol collection was carried out with the micro-cluster sampling device that was designed by the Academy of Military Medical Sciences (Beijing, China). Airborne biological particles were gathered using liquid impingers (All Glass Impinger 30, AGI30; ACE Glass Inc., Vineland, NJ, USA) that rely on inertial collection mechanisms. The flow rate was 12.5 L min^-1^, when the pressure drop across the orifice was 41 cm Hg. The tip of the capillary stem was situated 30 mm from the flask bottom; therefore, when filled with 20 mL of liquid, the nozzle outlet was 10 mm above the resting liquid surface. Airborne biological particles drawn into the 1 mm diameter nozzle and down the capillary stem, then impacted and penetrated the liquid surface [32]. The adhesion properties involving liquid and airborne particles were exploited by this technology to capture the microorganisms [33].

### Simulation experiment

#### Biosecurity level

All assays were conducted in a Biosafety Level 3 setting. The containment laboratory – Biosafety Level 3 – was designed for work with Risk Group 3 microorganisms in large volumes and Risk Group 2 microorganisms at high concentrations. In addition, the Biosafety Level 3 containment laboratory was equipped with a negative pressure isolation unit, which was used in our study for simulating accidents during laboratory procedures.

#### Normal operation of laboratory procedures

Normal laboratory procedures were carried out under controlled conditions for the purpose of monitoring the amount and character of aerosol produced. In group I (zootomy), chickens diagnosed with AIV upon autopsy were sectioned and the excised tissues frozen at −20°C. The total collection time was 1 h. Disinfection of the anatomical units after collection and air sampling took an additional 1 h. Control air samples were taken in the same units one day later. This process was repeated a total of six times, and six chickens were necropsied. In group II (grinding), the frozen lung tissues were thawed, ground in a mortar, and transferred to a centrifuge tube for storage at 4°C. Aerosol collections were carried out during grinding (at 20 min into the procedure) and 10 min after the homogenate was collected, for a total of 30 min. After disinfection of the area, air samples were immediately collected. This entire process was repeated six times. In group III (centrifugation) the tissue homogenates were centrifuged at 5000 rpm for 20 min at 4°C. Air samples were collected from the point of initial handling of the centrifuge tube to the centrifuge to the end of the spin when the centrifuge instrument was opened and the tube removed. To balance the centrifuge for each spin, the six homogenates were processed in groups of two, so that the experiment was repeated three times. Air sampling was performed after disinfection for use as the control. Collection times were 30 min. In group IV (pipetting), the supernatant resulting from the centrifugation step was transferred by a pipettor to a new tube, using blow-out and pull-in processes a total of 10 times to mix each sample. To ensure continuity of the experimental integrity, the pipetting experiment was carried out in another similar laboratory room that did not communicate with the centrifuge room. The same air sampling procedure was carried out for 30 min during the experiment and after disinfection. Similar to group III, the procedures of group IV were repeated three times. In group V (magnetic stirring), the virus-containing supernatant samples were diluted with PBS (pH 7.2) in a 10-fold series. This occurred by taking 1 mL of supernatant and adding to PBS in a 9 mL beaker with a stir bar that was placed on an active magnetic stirrer. Air sampling occurred throughout the entire process and after disinfection of the space, three times at 30 min each. In group VI (egg inoculation), 10 chick embryos (about 9-days-old) were inoculated with 0.2 mL diluted virus sample (10-fold diluted virus stock solution) per embryo. Air samples were collected throughout the process, including the time when the virus was drawn into the syringe, air bubbles were removed from the syringe, chick embryos were injected, and sealing wax was applied. A total of 10 chicken embryos were inoculated in each air monitoring experiment. Air samples were collected throughout and after disinfection, taking 30 min. The procedure was repeated three times, requiring a total of 30 chick embryos to be inoculated. In group VII (zoogenetic infection), five chickens (7-days-old) was intranasally administered virus at a dose of 0.2 mL per chicken. The zoogenetic infection experiments were repeated three times. Air sampling was carried out at intervals of 30 min each, and after disinfection.

#### Laboratory procedures with simulated accidents

Laboratory simulation of accidents that most frequently occur during the routine processing of influenza viruses were carried out inside a negative pressure isolation unit equipped with a simple robotic arm and operating gloves. In accident group I (broken glass containers), glass containers holding influenza virus suspensions were dropped in a free-fall and the broken pieces collected as in a routine clean-up procedure. In accident group II (syringe-ejected influenza virus suspension), 3 mL of the virus dilution (10-fold diluted virus stock solution) was drawn into a disposable syringe, and the plunger was depressed to spray out a small amount of the virus suspension into the air. In accident group III (centrifuge tube rupture), empty microcentrifuge tubes were centrifuged at inappropriate speeds to produce cracked tubes. Then, 1 mL of virus solution was added to the tube and the tube was closed and manually squeezed until the tube ruptured and the liquid splashed out, at which point air samples were collected. All of the processes for the three groups were repeated five times. For each, air samples were collected before the procedure for use as the control. The collection time was 30 min.

### Detection

**Figure 5:**
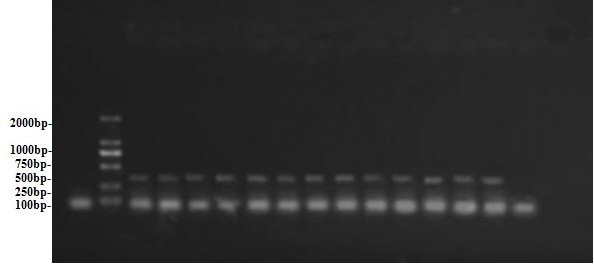
**Rt-PCR analyses of virus adsorb-proliferation sensitivity.** Lanes: 1 negative; 2 DNA markers 3–16 Rt-PCR results of 10-fold diluted series of virus solution.

After virus propagation in chicken eggs, the allantoic fluid was collected and processed according to standard procedures for detections of AIV by Rt-PCR and HA text, both detections were repeated twice. The sensitivity of the Rt-PCR procedure to detect AIV was evaluated by using a 10-fold diluted virus series; AIV was detectable by this method up to 10 [13] dilution of the virus solution (Figure 5).

#### Treatment of liquid impingement samplers

The processing of samples collected by the air monitor was based on well-known hemagglutination properties of H5N1 [34]. Briefly, the virus in each sample would bind to RBCs, causing an agglutination effect that was proportional to the virus concentration. In addition, the samples were used to inoculate 9-day-old embrocated chicken eggs. Eggs were incubated for 72 h at 37°C and then chilled overnight at 4°C. The allantoic fluid from each egg was collected separately and detected by standard Rt-PCR. For long-term storage, chicken RBCs (CRBCs) were treated with formaldehyde.

*Treatment of erythrocytes.* Chicken blood was divided into several tubes and centrifuged at 3000 rpm for 10 min; the resultant supernatant was carefully removed by pipetting and mixed with sterile PBS (pH 7.2). Three PBS washing steps by centrifugation were carried out. Finally, the erythrocyte sediment was diluted with PBS at a 1:20 (vol/vol) ratio and 2.5% glutaraldehyde was added at a 4:1 (vol/vol) ratio and the solution was oscillated at room temperature for 45 min. The treated erythrocytes were washed four times with PBS by centrifuging (3000 rpm for 5 min each), and finally resuspended in PBS at 2% (treated erythrocytes/ volume of PBS) and stored at 4°C until use.

*Treatment of sample.* Samples collected from experimental groups and their respective controls were incubated on ice to chill. Then, treated erythrocytes were added at a 1:2.5 (vol/vol) ratio. Adsorption was allowed to proceed for 1 h at 4°C, during which time the sample was intermittently mixed by inverting the tubes several times. After the incubation, samples were centrifuged at 5000 rpm for 10 min at 4°C, and the supernatant was discarded. The sediment was resuspended with 1 mL PBS on ice and recentrifuged at 5000 rpm for 10 min at 4°C, after which the supernatant was discarded. The sediment was then resuspended in a mixture of 0.1 mL amb-antibacterial media (supplemented with penicillin and streptomycin, 100 U/mL each) and 0.1 mL PBS. The mixture was incubated for 1 h at 37°C with intermittent mixing by inverting the tubes several times. The entire volume (0.2 mL) of each sample was then used to inoculate a 9-day-old embryonated chicken egg. The inoculated eggs were incubated for 72 h at 37°C and then chilled overnight at 4°C. The allantoic fluid from each egg was collected, except in the cases where the embryo had died within 24 h after inoculation.

#### Reverse-transcription PCR and HA text

A standard Rt-PCR test was performed to confirm the presence of the virus in allantoic fluid. A total of 0.2 mL of the allantoic fluid was used for RNA extraction by the Qiagen RNeasy Mini Kit (Cat No. 75182) and then applied to a reaction mix using reagents from the one-step Rt-PCR amplification Mini Kit (Qiagen Cat No. 210212), as follows: initial heating step, 95°C for 15 min; reverse transcription, 55°C for 30 min; initial PCR activation step, 95°C for 15 min; 35 cycles of amplification (denaturation, 94°C for 1 min; annealing, 60°C for 1 min; extension, 72°C for 1 min); final extension, 72°C. Gene-specific primers for the NP gene of H5N1 were: NP-Forward, 5′-GCATTGTCTCCGAAGAAATAAG-3′ and NP-Reverse, 5′-CAGATACT GGGCHATAAGRAC-3′. The expected length of the amplicon was 320 bp.

The allantoic fluid (positive by Rt-PCR) was detectable by hemagglutination test that performed to confirm the presence of the virus. Hemagglutination test was performed in 96-wells microtiter plates with 1% CRBCs.

## Abbreviations

Rt-PCR, Reverse-transcript polymerase chain reaction; HA, Hemagglutination; GLP, Good laboratory practice; WHO, World Health Organization; AGI30, All Glass Impinger 30; CRBC, Chicken red blood cell.

## Competing interests

The authors declare that they have no competing interests.

## Authors’ contributions

ZL carried out most of the experiments and wrote the manuscript. JL, ZX and ZL designed the equipment. ZD, DL, LM and LL participated in the planning of the project. DL, LM and LL collected the samples. ZL and ZD performed the Rt-PCR, analysis and interpretation of data. FG and ZX conceived of the study and participated in its design and coordination. All authors read and approved the final manuscript.
